# Changes in Vitellogenin (Vg) and Stress Protein (HSP 70) in Honey Bee (*Apis mellifera anatoliaca*) Groups under Different Diets Linked with Physico-Chemical, Antioxidant and Fatty and Amino Acid Profiles

**DOI:** 10.3390/insects13110985

**Published:** 2022-10-26

**Authors:** Aybike Sarioğlu-Bozkurt, Erkan Topal, Nazmiye Güneş, Engin Üçeş, Mihaiela Cornea-Cipcigan, İlknur Coşkun, Lucian Cuibus, Rodica Mărgăoan

**Affiliations:** 1 Department of Biochemistry, School of Veterinary Medicine, Bursa Uludag University, Nilüfer, 16059 Bursa, Turkey; 2 Izmir Food Control Laboratory Directorate, Bornova, 35100 Izmir, Turkey; 3 Apiculture Research Center, Aegean Agricultural Research Institute, 35660 Izmir, Turkey; 4 Faculty of Horticulture and Business in Rural Development, University of Agricultural Sciences and Veterinary Medicine Cluj-Napoca, 400372 Cluj-Napoca, Romania; 5 Advanced Horticultural Research Institute of Transylvania, University of Agricultural Sciences and Veterinary Medicine Cluj-Napoca, 400372 Cluj-Napoca, Romania; 6 Altıparmak Gıda Sanayi ve Ticaret A.Ş., 34782 Istanbul, Turkey; 7 Department of Food Science, University of Agricultural Sciences and Veterinary Medicine Cluj-Napoca, 400372 Cluj-Napoca, Romania

**Keywords:** vitellogenin, HSP 70, amino acids, fatty acids, physico-chemical composition, multivariate analysis

## Abstract

**Simple Summary:**

Honey bee health, longevity and colony development depend on the quantity and quality of nutrients stored in the hive. In honey bees, protein feeding modulates both individual and social immunity. The present study aimed to determine the correct feeding model by examining the changes in vitellogenin (Vg) and heat shock (HSP 70) proteins affecting honey bees’ stress and overwintering ability before and after wintering. Vg levels in the hemolymph of nurse bees were higher than in forager bees in the samples taken after feeding. Increased values in amino acids and fat percentage (%) were correlated with the increased HSP 70 value in forager bees fed mixed pollen in early spring compared with the HSP 70 level in nurse bees, which gradually decreased. Therefore, nutritional quality and diversity can positively affect the health of honey bees.

**Abstract:**

Honey bee colonies are often subjected to diseases, nutrition quality, temperature and other stresses depending on environmental and climatic conditions. As a result of malnutrition, the level of Vg protein decreases, leading to overwintering losses. The Vg values must be high for a successful wintering, especially before wintering. If good nutrition is not reached, the long winter period may cause an increase in colony losses. Supplementary feeding is essential for colony sustainability when floral resources are insufficient, as in recent years with the emerging climate changes. Furthermore, quality food sources or nutrients are significant for maintaining honey bee health and longevity. This study examined the changes in HSP 70 and Vg proteins in 6 groups of 48 colonies fed with five different nutrients. The fatty acids that are present in the highest amount in *Cistus creticus* (Pink rock-rose), *Papaver somniferum* (Opium poppy) and mixed pollen samples were linoleic, palmitic and cis-9-oleic acids. The highest values in proline, lysine and glutamic acid were determined in *C. creticus* pollen. Regarding the *P. somniferum* pollen, the highest values were observed in lysine, proline, glutamic and aspartic acids. The highest values in lysine, proline, leucine and aspartic acid were noticed in mixed pollen. The effect of different feeding on Vg protein in nurse and forager bee samples was higher in the mixed pollen group in the fall period. In nurse bees, the mixed pollen group was followed by *Cistus creticus* pollen > *Papaver somniferum* pollen > sugar syrup > commercial bee cake > control group, respectively (*p* < 0.05). In forager bees, the order was mixed pollen, *P. somniferum* pollen, *C. creticus* pollen, commercial bee cake, sugar syrup and control. In the early spring period, the Vg levels were high in the mixed pollen group in the nurse bees and the commercial bee cake group in the forager bees. In the fall period, the HSP 70 value of the forager and nurse bees was the lowest in the *C. creticus* group (*p* < 0.05). In early spring, the active period of flora, a statistical difference was found between the treatment groups.

## 1. Introduction

Honey bee health, longevity and colony development depend on the quantity and quality of nutrients stored in the hive [1]. Honey bees meet their nutritional needs with nectar and pollen from the natural flora [2]. Basically, honey bees obtain carbohydrates from nectar and all other nutrients, especially proteins, from pollen [3,4]. The nutrition of the honey bee colony depends on the storage of nectar as honey, and pollen as bee bread [5]. Nurse bees are the feeding center of the colony, consuming pollen and feeding all other colony members [6]. Honey bees’ nutrition involves converting pollen and nectar into colony stores and then into brood feed in the form of royal jelly [7]. The most significant proteins belong to the Major Royal Jelly Protein (MRJP) family secreted by the hypopharyngeal glands of nurse bees taking care of the brood in the hive, characteristic of *Apis cerana* and *A. mellifera*, respectively [8]. Forager bees’ protein needs and intake are influenced by digestion, intestinal absorption, worker bee age and the colony’s division of labor effectiveness. While the nurse bees digest the protein, they also provide nutrition to the queen bee [9].

Beekeeping is an agricultural activity that contains many stresses and risks for the beekeeper’s colony losses [10,11]. The risks to which the honey bee are exposed are high, such as climate, flora and incorrect beekeeping practices [12]. In order to survive, the internal environment must be in balance and adapt to the increased changes in environmental conditions. The concept of stress and stress response is a valuable approach to understanding bees’ physiological and behavioral responses in recent years [13]. Stress can reduce pollen collection performance in honey bees. A controlled non-pathogenic stressor (immune challenge) is known to cause a change in the forage preferences of bees, reducing the bee’s pollen foraging and prolonging its foraging time in flowers. Stress also reduces the amount of octopamine in the forager’s brain, which is involved in the regulation of foraging and flight behavior [14]. It has been reported that stress can be particularly detrimental to the performance of forager bees and that stressed bees prefer nectar-rich sources [15]. Stress, even at low levels, can have significant negative consequences on both foraging behavior and the nutritional balance of the colony [16]. Research shows that there is a relationship between nutrition and stress. Feeding patterns are known to initiate, enhance, or even increase the susceptibility of the stress response. For example, feeding pattern sheds light on the mechanism of aggression and behavior shown by bees during seasons with insufficient nectar flow [17]. At the same time, the effort to protect the products they produce also contributes to this aggressive behavior. Providing appropriate food and water is a strategy to help the body recover [18,19]. In honey bees, protein feeding alters both individual and social immunity. 

The study conducted by Alaux et al. (2010) evaluated the individual immune parameters (hemocyte concentration, fat body content and phenoloxidase activity) and social immunity parameter (glucose oxidase (GOX) activity) of dietary protein amount (monofloral pollen) and diet diversity (polyfloral pollen) in honey bees (*Apis mellifera*). The results showed that polyfloral diets and protein-rich diets resulted in higher GOX activity compared to monofloral diets. These results reveal a connection between protein nutrition and immunity in honey bees [20]. 

Vg is a phosphoglycoprotein with a molecular weight of 180 kDa, synthesized from fat body. Vg is a female-specific glucolipoprotein yolk precursor produced by all oviparous animals. Vg is associated with egg production in queen bees and has functions that protect honey bees against oxidative stress and increase their life span. It is also known to influence development, as it provides the development of a fat body [20,21,22,23,24,25]. The synthesis of Vg in the honey bee depends on the nutritional status and especially on the availability and quality of pollen, because pollen is the only naturally occurring source of amino acids for honey bees [26]. Very high levels of Vg have been detected in the hemolymph of honey bee queens. It has been determined that this level varies in workers depending on the task distribution [23]. For example, high Vg levels have been reported in nurse bees working inside the hive and low levels in forager bees working outside the hive [27,28,29]. These differences in Vg levels are related to the different immune competence of worker honey bees, the class they are assigned to (e.g., nurse and forager bees) and the variation observed in the lifespan of different worker types [30]. At the same time, an important environmental variable affecting worker honey bees’ age-based division of labor is their nutritional status [31]. Heat shock proteins (HSPs) are a group of proteins found in cells of all life forms. Increases in the synthesis of these proteins can occur when a cell is exposed to heat or cold when it encounters various environmental stresses such as oxygen, nutrient or water deprivation, or in the case of various drug applications. The protein with a molecular weight of 70 kDa, known as HSP 70, is used as a molecular marker to determine the cellular and whole-organism stress [32,33].

A microarray study of brain tissues of various bee species, primarily *Apis mellifera*, showed that differential expression of several 70 kD and 80 kD stress proteins contribute to species-specific stress tolerance [34]. Since the HSP 70 values measured from the chest of the bee were not induced by stress in bees exposed to high temperatures in previous studies, many studies used brain tissue with a stress level six times higher than the breast tissue for stress parameter measurement [35]. 

In this study, different feeding models were applied to the colonies formed with equal strength at the beginning of the trial (in September) in the autumn period. It is aimed to reveal the possible effect of feeding by examining the changes in parameters (Hsp, Vg) with bee samples taken before wintering and in early spring. Furthermore, differences in feeding product types concerning pollen samples (i.e., fatty and amino acids, physico-chemical composition and antioxidant activity) were evaluated by principal component analysis (PCA) and heat mapping in order to assess their effects on forager and nurse bees’ Vg and HSP 70 protein levels in different seasonal periods.

## 2. Materials and Methods

The study was conducted in the apiary of Aegean Agricultural Research Institute Directorate located in the Menemen District of Izmir Province (N 38°33′54″ E 27°3′27″). In the experiment, sister queens of the Efe Bee (*Apis mellifera anatoliaca*) were used due to the breeding work carried out by the Aegean Agricultural Research Institute produced in 2020. Colonies were formed from 3 frames and 1 kg package bees on 14 September 2020. The experimental groups consisted of 6 groups and a total of 48 colonies, including the control group, sugar syrup group, *C. creticus* pollen group, *P. somniferum* pollen group, mixed pollen group and commercial bee cake group, with 8 colonies in each group. Feeding was supplementary, and the experiment was conducted in hives under natural environmental conditions. The trial began when the natural flora was insufficient.

In the study, *P. somniferum* pollen, an industrial plant, *C. creticus* pollen as a natural flora source, mixed spring pollen (as evaluated by the beekeeper) and sugar syrup made from beet sugar were used. The pollen obtained from the producers was stored in a deep freezer until use. Commercial bee cake (composition: powdered sugar, invert sugar syrup) was selected from the products available in the market. While fresh pollen was used for the colony’s protein requirement, a beet sugar–water mixture was used to meet the carbohydrate requirement. In order to ensure the freshness of the pollen and to easily observe the rate of consumption and storage in the colonies, it was provided to the colonies according to the consumption status. The pollen types were moistened with sugar syrup and given to colonies in the form of meatballs. The colony was fed with a 2:1 sugar–water mixture to form honey stores. The control group was fed 1 L of sugar syrup to eliminate the stress on the first day. The first feeding was carried out on the first day the experimental material was created. The research was planned according to the Randomized Plots Experimental Design. In order to determine the effects of feeding on colony performance between the groups, honey bee samples were collected from the hives in late fall as the first period and after overwintering (early spring) in the second period.

### 2.1. Stress Protein Analysis (HSP 70)

Within the groups to be formed, 10 nurse and 10 forager bee samples were taken from each of the 8 hives, placed in a deep freezer at −20 °C, and stored for the removal of bee brains. Brain tissue was removed from the bee samples under a microscope and on dry ice and transferred to Eppendorf tubes. The brain tissue in these tubes was homogenized by thoroughly crushing with PBS-azide-TAME buffer, then centrifuged at 4 °C for 20 min at 13,000× *g*. Total protein values were measured by protein assay kit (#5000112, Bio-Rad, Hercules, CA, USA) in the supernatant. HSP 70 values in 2000 ng total protein were read, necessary dilutions were made according to the total protein values, and the concentrations of the samples were calculated according to the Standards [33,36]. The standard used was for coated ELISA H9776-Heat Shock Protein 70 from bovine brain (Sigma-Aldrich, Inc., St. Louis, MO, USA). The primary antibody we used for coated ELISA was H5147-Sigma-Aldrich Monoclonal Anti-Heat Shock Protein 70 antibody, and the secondary antibody was Goat Anti-Mouse IgG (H + L)-HRP Conjugate #1706516 [36].

### 2.2. Vg Protein Analysis

In this study, the ELISA method measured the Vg levels of 10 foragers and 10 nurse bees taken from each hive in different feeding groups. For hemolymph extraction, the study of Mayack and Naug was taken as an example [37]. The bees were freeze-killed, and their mouths glued to avoid any possible contamination of the hemolymph. Afterward, the distal end of the antennae was clipped with scissors and each bee was placed upside down in a centrifuge tube at 16,000× *g* for 30 s. Hemolymph dripped from the ends of the antennae, and 2 mL of the hemolymph was diluted with 58 mL of distilled water. It was kept at −20 °C until the day of measurement. After providing the necessary bee samples, Vg values were determined in the hemolymph according to the Vg sandwich ELISA kit (VTG-MBS 284624, MyBioSource, San Diego, CA, USA) produced for honey bees by applying all procedures of the commercial kit procedure. Since no honey-bee-specific vitellogenin ELISA kit could be found, a kit specific to *Nasonia vitripennis*, its closest relative, was used.

### 2.3. Essential Amino Acids

The Ezfaast (GC-MS) analysis kit method for Modified Protein Hydrolysates was used. Ground pollen (0.1 g) samples were weighed and microwaved with 8 mL of 6 N HCL (hydrochloric acid) and processed. Samples were placed into the microwave chamber (CEM, Mars 6) at 200 °C for 20 min at a power of 1800 watt. After pre-treatment according to the Ezfaast (GC-MS) analysis kit method for the hydrolyzed sample Protein Hydrolysates, the obtained extracts were analyzed via GC–MS. The GC-MS system used was the Agilent 5975 GC-MS device. The column installed in the gas chromatograph was a Phenomenex Ezfaast AAA Column Capillary (10 m × 0.25 mm × 0.25 µm film thickness). Helium was used as the carrier gas. The inlet temperature was 250 °C and for the column was 110 °C initial temperature increased by 30 °C/min to 320 °C. The injected volume was 2.5 µL. The electron impact (EI) mass spectra were recorded at an ionization energy of 70 eV and a source temperature of 240 °C. Identification of the amino acids was accomplished by comparing their retention times with known standards (Phenomenex EZfaast GC-MS, 200 nmol/mL). The amount of amino acid components was expressed as mg/g [38]. 

### 2.4. Fatty Acids

Ground pollen samples (10 g) were taken, pre-treated and analyzed by methylation of fatty acids using an Agilent 5975 GC-MS device. Acid hydrolysis and lipid extraction were performed for pollen samples. The lipids were extracted with hexane, and then transferred to the container where esterification was carried out and the hexane was evaporated. The remaining amount of lipids were subjected to a minimum of 200 mg methylation process and analyzed. Before, calibration was carried out with fame mix standards. Esterification processes were carried out for the remaining part of the extracted lipids. For esterification of fatty acids, 2 mL of esterification reagent (mixture of 2.75 mL of concentrated hydrochloric acid and 47.25 mL of methanol) was added after lipid extraction and vortexed. It was incubated in an oven at 100 °C for 40 min, then the samples were cooled and 2 mL of deionized water was added. Afterwards, esterified fatty acids were extracted by adding 2 mL of hexane; the hexane supernatant was taken into the vial and injected into the GC-MS system. Fatty acids were determined quantitatively with the GC-MS system. The GC-MS system used was the Agilent 5975 GC-MS device. The column installed in the gas chromatograph was a DB-23 (60 m × 0.25 mm × 0.15 µm film thickness). Helium was used as the carrier gas. The inlet temperature was 250 °C and for the column was: 50 °C initial temperature and hold for 1 min, then increased by 25 °C/min to 175 °C, and afterward increased by 4 °C/min to 230 °C and hold for 5 min. The injected volume was 1.0 µL. The electron impact (EI) mass spectra were recorded at an ionization energy of 70 eV and a source temperature of 230 °C. Identification of the fatty acids was accomplished by comparing their retention times with known standards (FAME mix standart—Supelco 37 Component FAME Mix CRM47885). The amount of fatty acids was expressed as mg fatty acids/100 g pollen/bee cake [39,40,41]. 

### 2.5. Physico-Chemical Composition and Antioxidant Activity

The methods for physico-chemical composition determination (ash%, moisture%, fat%, protein%, fructose%, glucose% and fructose/glucose ratio) and antioxidant activity assay were previously presented [42]. 

### 2.6. Statistical Analysis

Statistical significance was evaluated by one-way analysis of variance (ANOVA) and post hoc Tukey test with XLSTAT 2021.2. software (Addinsoft, New York, NY, USA), where the independent variables are the different food sources used and the dependent variables are the general conditions of the forager and worker bees’ Vg and HSP 70 protein levels in different seasonal periods. PCA was used to show the similarities and differences between different feeding products in foragers and nurse bees and changes in Vg and HSP 70 levels. Heatmap and dendrograms were performed using the Euclidean distance with complete linkage to highlight the differences in feeding product types concerning pollen samples (i.e., fatty and amino acids, physico-chemical composition and antioxidant activity) and to assess their effects on forager and worker bees’ Vg and HSP 70 protein levels in different seasonal periods. 

## 3. Results

### 3.1. Fatty Acids

The fatty acid profiles of the food sources used in the experiment are given in Table 1.

Table 1 shows that no fatty acids were detected in commercial bee cakes. *C. creticus* pollen presented the highest content in linoleic acid, followed by palmitic, cis-9-oleic and cis-13-16-docosadienoic acids. The lowest levels of fatty acids were noticed in cis-11-14-eicosadienoic and cis-8-11-14-eicosatrienoic acids. *P. somniferum* pollen presented the highest level of palmitic acid, followed by linoleic, cis-9-oleic, stearic and linolenic acids. The lowest level was noticed in cis-8-11-14-eicosatrienoic acid, followed by lignoceric, cis-13-16-docosadienoic and cis-11-14-eicosadienoic acids. Even though present in low levels in *C. creticus* pollen, undecanoic and arachidonic acids were absent in *P. somniferum* pollen. Mixed pollen contained increased values in palmitic, linoleic and cis-9-oleic acids, followed by lower levels in stearic, linolenic, euric, myristic and eicosadienoic acids.

### 3.2. Amino Acid Profiles

The amino acid profiles of the food sources used in the experiment are given in Table 2. The amino acid content presented the highest values in proline, lysine and glutamic acid in *C. creticus* pollen. Lower levels in leucine, aspartic acid, alanine, phenylalanine and histidine were also noticed. The lowest levels were shown in threonine, cysteine hydroxyproline and hydroxylysine. Regarding the *P. somniferum* pollen, the highest values were observed in lysine, proline, glutamic and aspartic acids, followed by leucine. Low levels in alanine, phenylalanine, glycine, histidine, valine and isoleucine were detected. Regarding mixed pollen, higher lysine, proline, leucine and aspartic acid values were noticed. The lowest values were shown in hydroxylysine, hydroxyproline, methionine, threonine, serine, valine and tyrosine. 

### 3.3. The General Condition of Colonies before and after Overwintering

Feeding studies were carried out considering the nectar sources, climatic conditions and wintering conditions in the region where the colonies are located. The amount of sugar syrup, pollen and commercial bee cake was given to the groups between 14 August 2020 and 24 February 2021 during the experiment, as shown in Supplementary Table S1. All the bee cakes (pollen and bee cake) were given in 2020. Feeding with pollen before overwintering positively affects overwintering ability and helps the colony’s development (especially in early spring). Throughout the experiment, it was observed that the bees in the pollen group were more vigorous, healthy and mobile (Supplementary Table S2).

**Figure 1:**
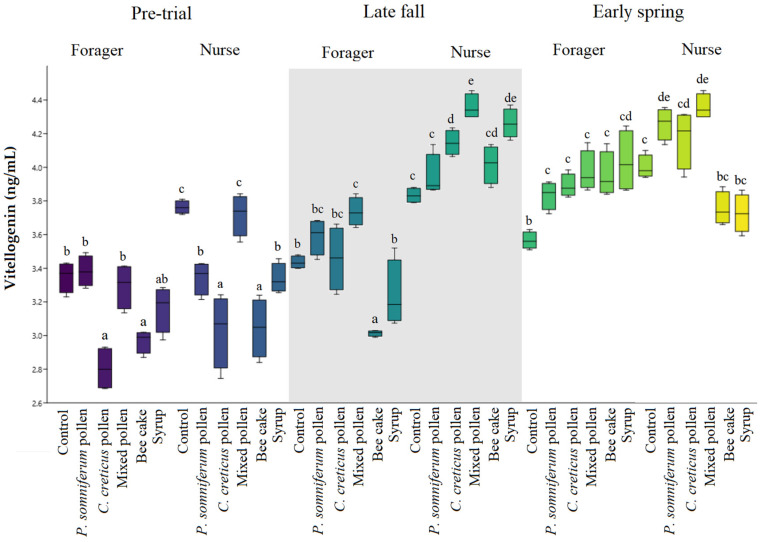
Vg content/values of groups in late fall (Period 1) and early spring (Period 2). Different letters denote significant differences (*p* < 0.05).

Hemolymph Vg values in all groups before feeding are given in Supplementary Figure S1, and Vg values in samples taken in late fall and early spring after feeding are shown in Figure 1. Since the experimental colonies were formed as a package, possible differences in the distribution of age groups may affect the different Vg levels in honey bee samples taken before the experimental groups. Vg levels were found to be close to each other in the bee samples taken before different feeding in both groups of nurses and forager bees, with higher values observed only in the commercial bee cake nurse bees’ group (Supplementary Figure S1), compared with the Vg levels in the hemolymph of nurse bees which were found to be higher than forager bees in the samples taken after feeding (Figure 1, late fall). Following the winter period, increased Vg values were noticed in nurse bees fed pollen (early spring), particularly in the mixed pollen group.

It was observed that the highest Vg value was obtained in the mixed pollen group in the nurse bee samples taken in the late autumn period (Period 1), and the order was mixed pollen > sugar syrup > *C. creticus* pollen > *P. somniferum* pollen > commercial bee cake > control group (Figure 1). In the late fall period (Period 1), the lowest value of the forager bees was noticed in the commercial bee cake group, with significant differences between the other feeding groups (*p* < 0.05). The nurse bees obtained the highest Vg value in the mixed pollen group in the second period (early spring period). In the second period of the forager bees, the same statistical significance was found between the control, *P.somniferum*, *C. creticus* and mixed pollen groups. 

**Figure 2:**
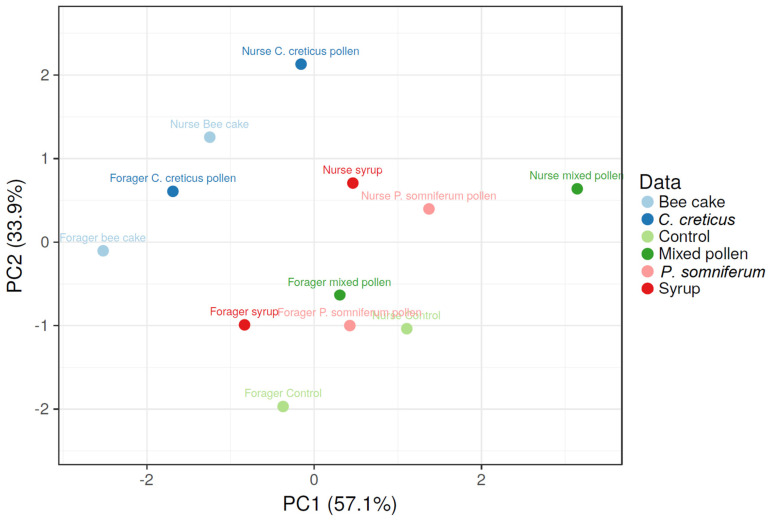
PCA was obtained according to Vg values of forager and nurse bees before feeding and after in late fall and early spring. The first two components explained 91% of the data variance. The variables with loading values close to zero present a similar pattern in the forager or nurse bees.

According to the PCA (Figure 2), the first quadrant (upper left) comprises the forager and nurse bees fed with *C. creticus* pollen that presented lower levels of Vg before feeding, which eventually increased in early spring and maintained its high level until late fall. The second quadrant (upper right) highlights the high content in Vg levels until fall in nurse bees that received the mixture of pollen, followed by the nurse bees fed syrup and *P. somniferum* pollen with high Vg values in early spring, which significantly decreased in late fall. In the next quadrant (lower right), the forager bees receiving mixed pollen and *P. somniferum* displayed lower Vg levels in spring and fall, respectively. The last quadrant (lower left) emphasizes the forager control and syrup-fed bees with low Vg levels until late fall.

When the pre-feeding HSP 70 values were examined, it was determined that although the values in the nurse bees in all study groups were slightly higher compared with the forager bees, significant differences were noticed only in the case of the *C. creticus* group nurse bees (Supplementary Figure S2).

**Figure 3:**
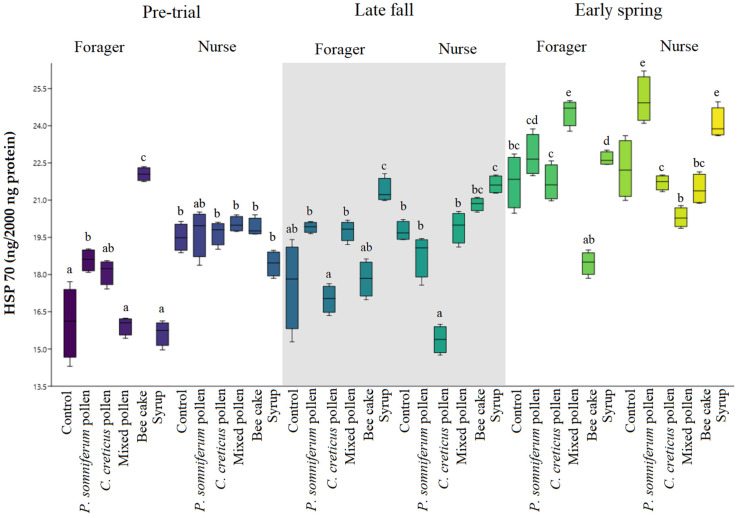
HSP 70 content/values of groups in late fall (Period 1) and early spring (Period 2). Different letters denote significant differences (*p* < 0.05).

When the study findings were examined regarding HSP 70 values for nurse bees (Figure 3), significant differences were found between the different products used in all study groups in the late fall (Period 1). In the forager bees, lower HSP 70 values were noticed in late fall compared with higher values in nurse bees (*p* < 0.05). HSP 70 values were the lowest in both forager and nurse bees in the *C. creticus* pollen group. In early spring (Period 2), there was a difference in *P. somniferum* pollen-fed nurse bees which exhibited increased HSP 70 values, as also seen in the syrup-fed group. Conversely, the other feeding groups noticed significantly lower levels in nurse bees. Forager bees showed higher levels in the mixed pollen group and lower levels in the commercial bee cake and control group. The commercial bee cake group showed minor stress in the second period. Since only sugar syrup feeding is conducted during this period, bees resort to the existing floral resources.

**Figure 4:**
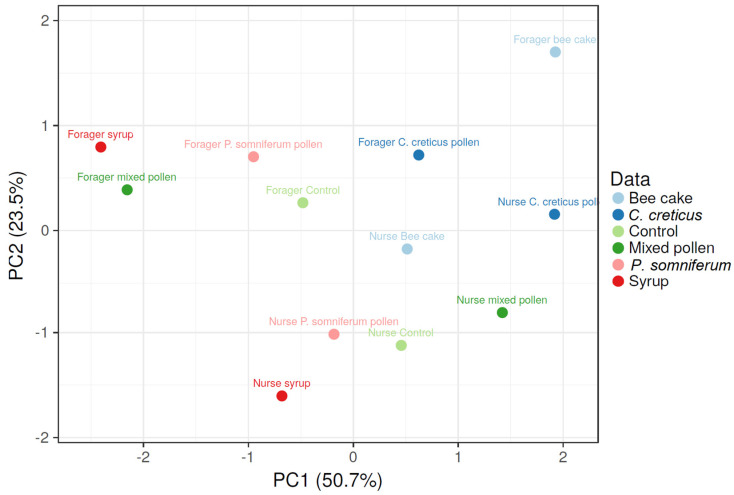
PCA was obtained according to HSP 70 values of forager and nurse bees before feeding and after in late fall and early spring. The first two components explained 74% of the data variance. The variables with loading values close to zero present a similar pattern in the forager or nurse bees.

According to the PCA (Figure 4), the lowest HSP 70 levels were shown in forager bees before treatment (fed mixed *P. somniferum* pollen or syrup), which significantly increased in early spring and slightly decreased in late fall (upper left quadrant). In forager bees in the control group, HSP 70 maintained its low levels until the end of the treatment. The following upper right quadrant highlights the bee-cake-fed forager bees with the lowest level in HSP 70, which slightly increased in early spring and maintained its level until late fall. In the following quadrant (lower right), no significant differences in HSP 70 levels were noticed in nurse bees fed mixed pollen. In control, the nurse bees had higher HSP 70 levels before feeding compared with low levels noticed in forager bees. The last lower left quadrant highlights the nurse bees fed with syrup and *P. somniferum* pollen with the highest HSP 70 levels in early spring, which was maintained until fall in syrup-fed bees, whereas HSP 70 in *P. somniferum* pollen-fed nurse bees significantly decreased in late fall.

### 3.4. Relationship between Feeding Patterns and Vg and HSP 70 Values according to Fatty Acid and Amino Acid Content

**Figure 5:**
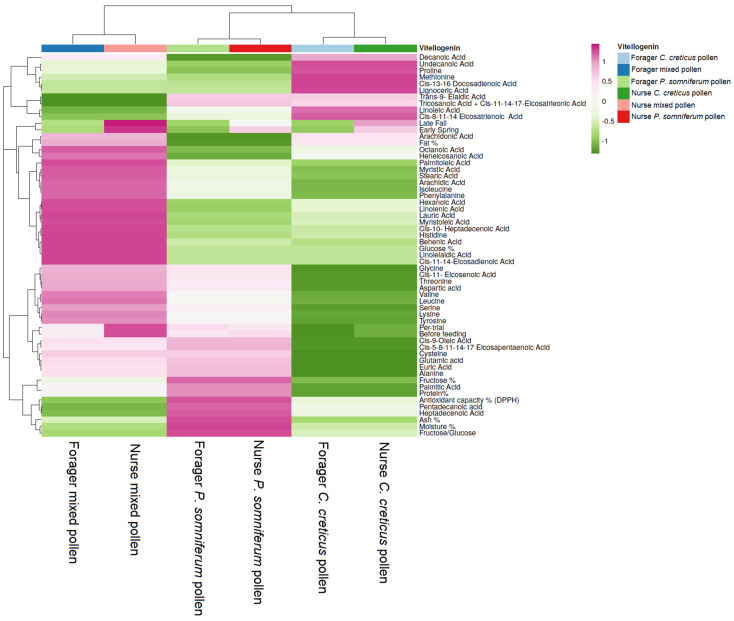
Hierarchical clustering and heatmap visualization of the Vg content present in forager and nurse bees based on the fatty and amino acid profiles of feeding product types. Columns indicate the product types fed to foragers and nurse bees and rows the products’ fatty acid and amino acid patterns along with the content in Vg in different periods. Cells are colored based on the quantity of Vg in each forager and nurse bee, where pink represents a strong positive correlation and green a strongly negative correlation. The row dendrogram resulted from the correlation between the fatty and amino acid profiles and Vg level in different feeding periods; the column dendrogram shows the correlation between foragers and nurse bees and feeding product types.

Hierarchical clustering and heat mapping were used to visualize similarities and differences between feeding product types and Vg values according to the fatty and amino acid content (Figure 5). Unambiguous discrimination between feeding products is seen by the different cluster positions of mixed pollen (first cluster), compared with the following *Papaver* (second cluster) and *Cistus* (third cluster) pollens. Forager and nurse bees fed mixed pollen were discriminated from the other bees fed different product types. Thus, following the importance score, in the first cluster, lower levels of trans-9-elaidic acid, tricosanoic acid + cis-11-14-17-eicosatrienoic acid, linoleic, pentadecanoic and heptadecenoic acids, and increased levels of palmitoleic, myristic acid, stearic and arachidic acids in amino acids isoleucine and phenylalanine were found along with a low Vg level before feeding. Regarding the forager bees, a low Vg level was noticed in early spring and late fall compared with an increased value in nurse bees. Furthermore, the amino acid content was higher than the *Papaver* and *Cistus* pollen, particularly threonine, valine, leucine, serine, lysine, tyrosine and aspartic acid. Lower levels in the antioxidant activity, ash%, moisture%, fructose–glucose ratio and amino acid profiles were observed in *Cistus* pollen-fed groups. Conversely, higher levels in fat%, octanoic and arachidonic acids and the Vg level in nurse bees in early spring and late fall were observed. The second cluster highlights the forager and nurse bees fed *P. somniferum* pollen with low levels in pentadecanoic and heptadecenoic acids and increased values in undecanoic acid and proline content. Regarding the forager bees fed *Papaver* pollen, a low level in Vg was noticed from early spring until late fall.

In contrast, a higher Vg level in nurse bees is noticed, especially in early spring, which decreased in late fall. In the third cluster, the forager and nurse bees fed *Cistus* pollen presented lower values in Vg before feeding pre-trial. Comparatively, in nurse bees, higher Vg levels were detected in early spring and late fall, also noticed in the other feeding groups, particularly in nurse bees fed mixed pollen. 

**Figure 6:**
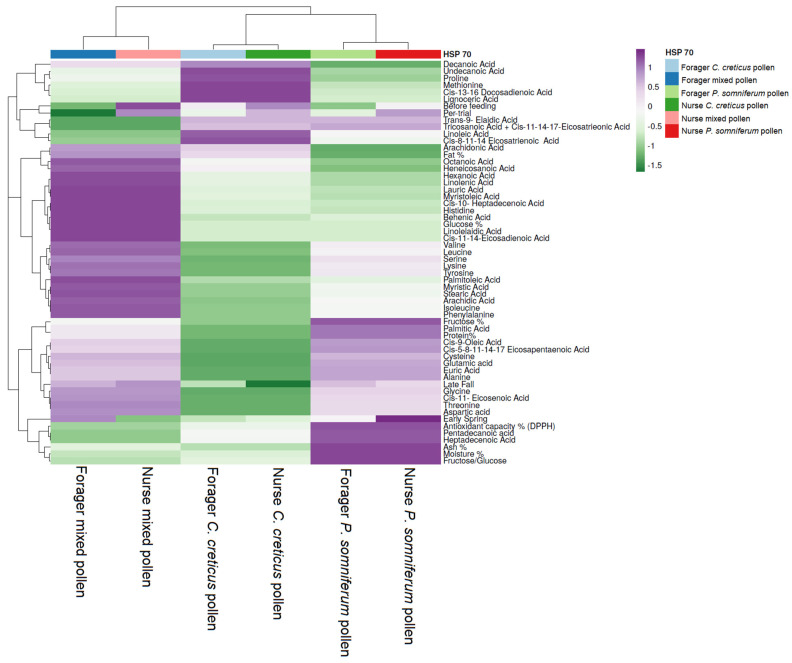
Hierarchical clustering and heatmap visualization of the HSP 70 protein content present in forager and nurse bees based on feeding product types’ fatty and amino acid profiles. Columns indicate the product types fed to foragers and nurse bees and rows the products’ fatty acid and amino acid patterns along with the content in HSP 70 in different periods. Cells are colored based on the quantity of HSP 70 in each forager and nurse bees, where purple represents a strong positive correlation and green a strongly negative correlation. The row dendrogram resulted from the correlation between the fatty and amino acid profiles and HSP 70 level in different feeding periods; the column dendrogram represents the correlation between forager and nurse bees and feeding product types.

Another HCA was performed to evaluate the relationship between the feeding pattern and HSP 70 content compared with the fatty and amino acid content of the pollen types used (Figure 6). The bees fed mixed pollen (first cluster) were differentiated from the *C. creticus* pollen (second cluster) and *P. somniferum* (third cluster), which clustered together. Thus, the first cluster highlights the decreased values in pentadecanoic acid, heptadecenoic acid, trans-9-elaidic acid and tricosanoic acid + cis-11-14-17-eicosatrieonic acid correlated to the deficient HSP 70 level in forager bees pre-trial. In contrast, the highest HSP 70 level was noticed in nurse bees before feeding compared with the other feeding groups. In addition, the increased amino acids and fat% content were correlated to the increased HSP 70 value in forager bees fed mixed pollen in early spring, whereas in nurse bees, the level gradually decreased. The antioxidant capacity, ash%, moisture% and fructose/glucose ratio negatively correlated with pentadecanoic and heptadecenoic acids. In the second cluster, the forager bees receiving *C. creticus* pollen exhibited an increased HSP 70 level which gradually increased starting from early spring and maintained until late fall, although slightly decreased. Regarding the fatty acid content, increased values in linoleic, cis-8-11-14-eicosatrienoic, undecanoic, cis-13-16-docosadienoic, lignoceric and decanoic acids were associated with the increased level in HSP 70 before feeding.

Conversely, low levels of HSP 70 in late fall, especially in nurse bees, were correlated to the amino acids, protein and fructose content in low amounts. In the last cluster, a slightly increased HSP 70 level was noticed in nurse bees in early spring as seen by the low presence of palmitic, cis-9-oleic, cis-5-8-11-14-17 eicosapentaenoic and euric acids and the amino acids alanine, cysteine and glutamic acid. In contradiction to *Cistus* pollen, the increased HSP 70 value in *Papaver*-fed bees in early spring may be due to the increased palmitic, cis-9-oleic, cis-5-8-11-14-17-eicosapentaenoic and euric acids and the amino acids alanine, cysteine and glutamic acid. 

## 4. Discussion

Our experiments show that colonies fed with pollen have Vg, Hsp 70, amino acid and fatty acid contents that offer the bee a higher overwintering potential. Previously, we demonstrated that the most preferred feed sources were *C. creticus* (25 g) and *P. somniferum* (24.8 g), the least preferred being mixed pollen. Furthermore, an increased lifespan of honey bees was noticed in *P. somniferum* fed honey bees, followed by the *C. creticus* pollen-fed group [42]. In different studies supporting our findings, honey bee colonies fed with natural feed emerged from winter stronger than those fed with protein supplements [43]. Yeninar et al. (2015) reported that feeding with two different pollen types and water during the pine honey period (fall) provided 80% better overwintering ability [44]. 

While the monofloral pollen diet does not cause any change in the immune system, a polyfloral diet supports the immune system [45]. In particular, the level of glucose oxidase activity in polyfloral diets was higher than in monofloral diets, demonstrating a correlation between protein, dietary diversity and the immune system in honey bees. The cells of brood and worker bees (nurse and forager) within a colony are influenced by available stored pollen, which is converted to Vg, the main stored protein in the fat body of nurse bees. Furthermore, the rise in Vg levels in nurse bees directly affects the size of the worker and brood populations at balance [31]. A study supporting our findings showed that nurse bees’ Vg levels were higher than forager bees [27]. Vg levels in the hemolymph of nurse bees were higher than in forager bees in the samples taken after feeding (Figure 1). In early spring, only sugar syrup was supplemented to the colonies, and they were allowed to benefit from the plant resources in the region. Additional feeding during this period is required for the colony’s future; in early spring, nectar and pollen sources support the development of colonies. The increased Vg level in the forager and nurse bees in the second period may be due to sample collection in early spring. During this period, there were suitable floral resources for colony development. This result is in line with previous studies that demonstrated similar levels of Vg for nurses and foragers of the same age [46]. 

The lack of floral resources before overwintering allowed the effect of feeding in the fall period to be seen in the colonies. Food quality has a positive effect on colony strength. The difference between colony developments increased with the enrichment of the flora in the early spring period. In support of our findings, seasonal, weather and temperature changes have great effects on the population dynamics of honey bee colonies and also on social organization [47], along with the effects of essential amino acid profile on behavior, colony health and longevity [48,49,50,51,52,53,54,55,56,57] and differences in plant preferences of honey bees [58]. In general, different levels of amino acid and fatty acid exist in different pollen sources [59]. This affects the foraging behavior of honey bees depending on the nutrient content. It makes it more challenging to fill the gaps in nutrient composition [60,61]. 

Studies report that polyfloral pollen sources are more effective than monofloral pollen sources for the survival of honey bees [45]. It was shown that Vg depends on the availability and quality of pollen [62]. Climate variability impacts nutritional factors related to the quality and quantity of nectar and pollen sources [63]. Temperature, rainfall and intensity of solar radiation have been associated with the foraging activities of honey bees [63,64,65,66]. Vg plays a role in immunity, antioxidation and lifespan beyond reproduction [67]. One study identified three Vg-differentiated genes (Vg-like-A, -B and -C) composed of Vg-like genes and protein structures and modifications. These genes were present in long-lived winter bees and summer bees with average life expectancy, but only Vg-like-A showed high expression in winter bees, as in Vg. In addition, Vg-like-A responded strongly to inflammatory and oxidative conditions, whereas Vg-like-B responded more to oxidative stress than Vg [68]. 

Challenges to bee health are multifactorial and include malnutrition, heat stress, agrochemicals and pathogens. The impact of heat stress is a relatively minor factor in current bee declines compared to agrochemicals and pathogens. However, heat stress harms bees’ foraging activity, pollination services, task-related physiology, immune competence, reproductive capacity, growth and development [15]. The colony adapts to organize the most suitable environmental conditions for itself against heat and cold stress. When environmental temperature changes, heat production is adjusted by regulating bee density due to migration activity and by the degree of endothermy [69]. Food and water shortages seriously affect the individual survival and population distribution of insects in nature [70]. In a recent study, biomarkers were used to assess changes in honey bee physiology pre- and post-hybrid lavender season in high and low weight gain colonies. Post-season increased levels of enzymes involved in antioxidant defenses (i.e., catalase, superoxide dismutase and glutathione peroxidase), acetylcholinesterase and decreased levels of alkaline phosphatase were associated with the loss of midgut homeostasis. Compared to low weight gain colonies, high weight gain colonies showed lower levels of almost all analyzed biomarkers [71]. Heat stress in honey bees (*A. mellifera*) has caused neural deficiencies that reduce short-term memory and impair the ability of adult bees to find food [72]. Because over-expression of stress proteins can be detrimental to an organism, environmental stressors should be reduced as much as possible by measuring gene regulatory mechanisms and the degree of heat shock response.

Bee physiology and tolerance to parasites vary depending on the type of pollen diet, and not only the availability of environmental resources but also their quality are essential [73]. The Vg level was higher in bees fed with pollen with high protein levels, and pollen with low or similar protein levels may have high Vg levels, which may be associated with high lipid content. In the pollens we used in our study, the highest protein content was found in mixed pollen, followed by *P. somniferum* pollen and *C. creticus* pollen. Regarding the levels of Vg, a higher level in the mixed pollen was noticed. These data are followed by *P. somniferum* pollen and *C. creticus* pollen, respectively. Lastly, the high lipid levels in *P. somniferum* pollen increased the production of Vg. The data we found are similar to the previous study in this sense [42]. Thus, beekeepers might supplement colony food to keep honey bees healthy and productive in times of food scarcity. It was demonstrated that protein feed with prebiotic and probiotic (FPP) supplementation proves to be efficient for the health of bees, particularly after 11 days of treatment, by enhancing their immune system and reducing stress levels in the long run [74]. In different studies, the HSP 70 gene expression peaked at midday and was correlated with the maximum temperature and the least amount of bee flight activity [75]. Given the many stressors honey bees face in the environment, in a different study, bees were fed honey, sucrose (SS), high fructose corn syrup (HFCS) or HFCS + pollen and then exposed to abiotic stress in the laboratory. Levels of stress proteins (SOD), heat shock protein (HSP 70) and potentially esterase were quantified to determine non-lethal stress. SOD levels were reduced in SS and HFCS treatments, indicating that these diets impair the response to oxidative stress, especially at 45 °C. HSP 70 levels were higher in the imidacloprid treatment, with no difference between control and temperature treatments. Diet significantly interacted with stress treatment with overall negative responses to HFCS. Higher worker mortality rates were reported in colonies fed SS. In cage and field trials, bees were reported to consume more honey and HFCS + P, meaning that additional carbohydrates contribute to non-lethal stress, and natural food sources are preferred by bees [76]. A similar study stated that giving 100 µg L-1 nano-zinc oxide to colonies during hot weather can increase the queen and worker bees’ performance and heat resistance (HSP 70) under hot climatic conditions [77]. It has been reported that laboratory and field studies may not give similar results, especially considering the stress factors of the honey bee; particularly in field studies, colonies may be under the influence of many stress factors [15,77].

## 5. Conclusions

Previous research reported a relationship between nutrition and stress initiated by diets which increase the stress response and sensitiveness of bees. Just as insufficient caloric intake weakens the human organism, it is most likely that honey bees might be subjected to diseases in stressful situations. At the same time, the contribution of the efforts to protect the product they produce is a new stress factor. For this reason, supplementary feeding is essential for the sustainability of the colonies when there have been insufficient floral resources in recent years, where climatic changes are experienced very frequently. The quality of the nutrients used in feeding has an important place in the health of the honey bee. The present study demonstrated that higher Vg values were noticed in the mixed pollen group in the nurse bee samples taken in the late autumn period.

Regarding the HSP 70, in early spring (Period 2), significant differences were noticed in *P. somniferum* pollen-fed nurse bees, as seen by the increased HSP 70 values. The same ascending trend was also seen in the syrup-fed group. While the correct feeding of the colonies, especially before wintering, affects the Vg level positively, other stress factors affecting the bees should not be ignored. While various stress factors in the colonies negatively affect the colony’s strength, the colony experiences a process leading to extinction. It should not be forgotten that supplemental feeding minimizes the stress level when disease might affect the colony and malnutrition, exposure to pesticides, strength of the colony, climatic conditions and other similar stress factors are evaluated as a whole. All these results give clues that nutritional quality and diversity can positively affect the health of honey bees.

## Figures and Tables

**Table 1:** Fatty acid composition of feeds in experimental groups (mg/100 g pollen or bee cake).

Fatty Acids	Commercial Bee Cake	*C. creticus* Pollen	Mixed Pollen	*P. somniferum* Pollen
Myristic Acid	nd	1.7 ± 0.1	7.5 ± 0.3	3.8 ± 0.6
Hexanoic Acid	nd	2.3 ± 0.1	6.6 ± 0.2	1.1 ± 0.5
Octanoic Acid	nd	1.2 ± 0.0	2.5 ± 0.5	0.4 ± 0.1
Decanoic Acid	nd	8.5 ± 0.3	6.2 ± 0.2	0.7 ± 0.2
Undecanoic Acid	nd	0.4 ± 0.0	0.1 ± 0.0	nd
Lauric Acid	nd	1.7 ± 0.2	5.0 ± 0.5	1.2 ± 0.2
Tridecanoic Acid	nd	nd	nd	nd
Myristoleic Acid	nd	1.5 ± 0.1	4.9 ± 0.6	0.8 ± 0.1
Pentadecanoic acid	nd	0.8 ± 0.0	0.6 ± 0.1	1.1 ± 0.3
Palmitic Acid	nd	204.4 ± 5.7	293.3 ± 8.8	355.5 ± 15.3
Palmitoleic Acid	nd	0.9 ± 0.3	6.9 ± 2.1	2.1 ± 0.1
Heptadecenoic Acid	nd	1.2 ± 0.1	0.9 ± 0.2	1.7 ± 0.3
Cis-10-Heptadecenoic Acid	nd	0.5 ± 0.0	5.5 ± 0.1	0.2 ± 0.1
Stearic Acid	nd	7.7 ± 1.1	25.9 ± 0.6	13.8 ± 0.5
Trans-9-Elaidic Acid	nd	0.6 ± 0.2	0.1 ± 0.0	0.6 ± 0.1
Cis-9-Oleic Acid	nd	36.7 ± 5.5	91.5 ± 2.7	102.8 ± 10.9
Linolelaidic Acid	nd	1.0 ± 0.1	1.5 ± 1.1	1.0 ± 0.2
Linoleic Acid	nd	352.1 ± 8.9	203.3 ± 11.3	267.8 ± 13.6
Arachidic Acid	nd	0.4 ± 0.0	1.1 ± 0.2	0.7 ± 0.2
Cis-11-Eicosenoic Acid	nd	0.9 ± 0.0	3.7 ± 1.1	3.0 ± 0.1
Linolenic Acid	nd	7.3 ± 0.2	9.7 ± 2.1	6.6 ± 0.3
Heneicosanoic Acid	nd	1.1 ± 0.0	1.6 ± 0.4	0.6 ± 0.0
Cis-11-14-Eicosadienoic Acid	nd	0.1 ± 0.0	0.4 ± 0.1	0.1 ± 0.0
Behenic Acid	nd	0.3 ± 0.0	1.5 ± 0.1	0.4 ± 0.2
Cis-8-11-14-Eicosatrienoic Acid	nd	0.1 ± 0.0	0.01 ± 0.0	0.04 ± 0.0
Euric Acid	nd	0.6 ± 0.1	3.6 ± 0.2	4.0 ± 0.1
Tricosanoic Acid + Cis-11-14-17-Eicosatrieonic Acid	nd	4.1 ± 0.3	1.7 ± 0.0	4.3 ±
Arachidonic Acid	nd	5.1 ± 0.2	6.3 ± 0.1	nd
Cis-13-16-Docosadienoic Acid	nd	17.2 ± 0.6	0.5 ± 0.0	0.1 ± 0.0
Lignoceric Acid	nd	1.3 ± 0.1	0.1 ± 0.0	0.1 ± 0.0
Cis-5-8-11-14-17-Eicosapentaenoic Acid	nd	1.9 ± 0.1	3.4 ± 0.0	3.8 ± 0.1
**Total**	**-**	**663.6**	**695.9**	**778.3**

Values are represented as mean ± standard deviation; nd: not detected.

**Table 2:** Amino acid content of feeds in experimental groups (mg/g).

	Commercial Bee Cake	*C. creticus* Pollen	Mixed Pollen	*P. somniferum* Pollen
Alanine	nd	8.1 ± 2.2	9.5 ± 0.6	9.7 ± 0.4
Glycine	nd	4.8 ± 1.2	7.2 ± 0.5	6.7 ± 0.7
Valine	nd	4.2 ± 0.9	5.9 ± 1.1	5.1 ± 0.6
Leucine	nd	10.6 ± 2.4	16.2 ± 3.3	13.3 ± 1.7
Isoleucine	nd	3.9 ± 2.2	5.9 ± 0.5	4.7 ± 0.5
Threonine	nd	1.6 ± 0.6	3.0 ± 0.7	2.6 ± 0.4
Serine	nd	2.5 ± 0.3	4.8 ± 2.9	4.0 ± 0.6
Proline	nd	33.9 ± 11.3	25.7 ± 5.1	21.9 ± 0.9
Aspartic acid	nd	9.7 ± 1.4	16.6 ± 4.4	14.7 ± 3.3
Methionine	nd	2.4 ± 0.2	1.6 ± 0.2	1.5 ± 0.2
Hydroxyproline	nd	0.4 ± 0.0	0.4 ± 0.1	0.4 ± 0.1
Glutamic acid	0.1	15.1 ± 0.5	20.3 ± 2.3	20.8 ± 4.3
Phenylalanine	nd	7.1 ± 0.3	11.4 ± 0.5	8.8 ± 2.3
Lysine	0.1	21.3 ± 0.6	31.9 ± 1.6	27.3 ± 5.1
Histidine	nd	6.8 ± 0.5	9.7 ± 0.4	6.5 ± 2.2
Hydroxylysine	nd	0.1 ± 0.0	0.1 ± 0.0	0.1 ± 0.0
Tyrosine	nd	3.1 ± 0.3	6.0 ± 0.5	4.8 ± 2.1
Cysteine	nd	1.8 ± 1.1	1.9 ± 0.2	1.9 ± 0.7
**Total**	**0.2**	**137.4**	**178.1**	**154.8**

Values are represented as mean ± standard deviation; nd, not detected.

## Data Availability

All data were stated in this manuscript.

## Supplementary Materials

The following supporting information can be downloaded at: https://www.mdpi.com/article/10.3390/insects13110985/s1, Table S1: Group Consumption During the Experimental Period; Table S2: Colony Numbers of the Groups at the Measurement Dates.
