# Supplementary material for: Changes in Vitellogenin (Vg) and Stress Protein (HSP 70) in Honey Bee (Apis mellifera anatoliaca) Groups under Different Diets Linked with Physico-Chemical, Antioxidant and Fatty and Amino Acid Profiles
Source: Insects. 2022 Oct 26;13(11):985. doi: 10.3390/insects13110985 (PMC9698881; doi:10.3390/insects13110985)
Supplement: Supplementary file 1 [file insects-13-00985-s001.zip › insects-1870542-Supplementary files.pdf]

Supplementary Table S1. Group Consumption During the Experimental Period

Groups	Sugar Syrup (lt)	Bee cake (gr)
Sugar Syrup	14.0	-
Commercial Bee Cake	14.0	1350
P. somniferum pollen	14.0	1350
C. creticus pollen	14.0	1350
Mixed pollen	14.0	1350

Supplementary Table S2. Colony Numbers of the Groups at the Measurement Dates

	Colony Numbers of the Groups					
Measurement	Control	Sugar Syrup	Commercial Bee Cake	*P. somniferum* pollen	*C. creticus* pollen	Mixed pollen
14.09.2020	8	8	8	8	8	8
18.11.2020	7	6	8	8	8	8
10.03.2021	4	6	7	8	7	7

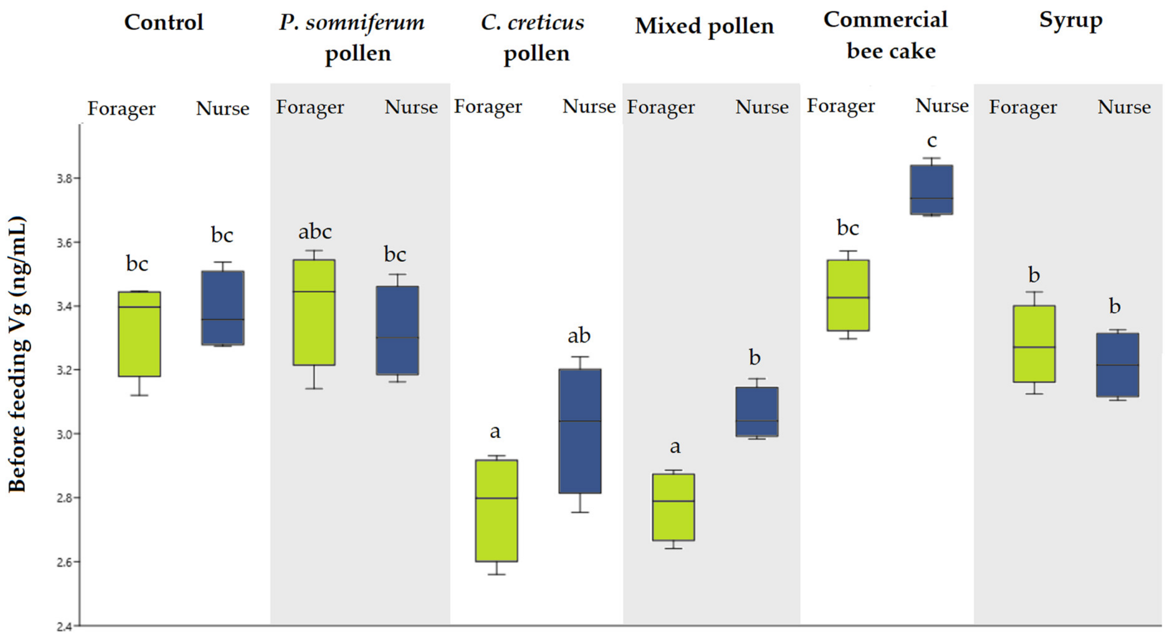

Supplementary Figure S1. Vg content/values of Groups before feeding. Different letters denote significant differences (p<0.05)

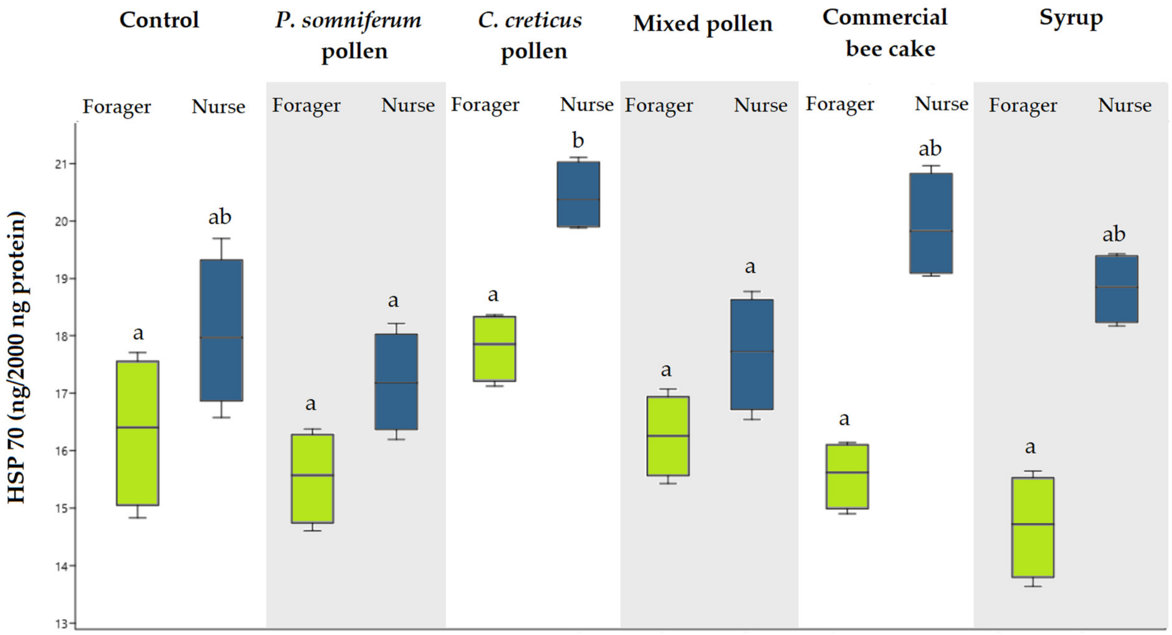

Supplementary Figure S2. HSP 70 content/values of Groups before feeding. Different letters denote significant differences ($p<0.05$)
